# Onsite clinic utilization and adherence in semiconductor employees at chronic disease risk

**DOI:** 10.1371/journal.pone.0321252

**Published:** 2025-04-24

**Authors:** Boyoon Choi, Kyungim Kim, Hyun Jin Park, Yun-Kyoung Song, Jung Mi Oh

**Affiliations:** 1 College of Pharmacy and Research Institute of Pharmaceutical Sciences, Seoul National University, Seoul, South Korea; 2 College of Pharmacy, Korea University, Sejong City, South Korea; King Abdulaziz University Faculty of Medicine, SAUDI ARABIA

## Abstract

**Introduction:**

The objective of this study was to evaluate the utilization and adherence of onsite clinics and identify the factors influencing them in semiconductor employees at risk of chronic diseases, including hypertension, diabetes, and dyslipidemia.

**Methods:**

A cross-sectional study was conducted through a retrospective review of electronic medical records from onsite clinics at a South Korean semiconductor company. The study focused on employees who visited the onsite clinics between 2013 and 2016 due to the risk of chronic diseases including hypertension, diabetes, and dyslipidemia. Descriptive statistics assessed clinic utilization and adherence, while multivariable logistic regression identified influencing factors, adjusted for age, sex, work type, work shift, workplace, specific onsite clinic, diagnosis, and disease duration.

**Results:**

Out of the 39,073 employees examined, 8,837 sought care at onsite clinics for managing chronic disease risks. The majority of these participants were male (88.2%) and predominantly aged in their 30s and 40s (84.6%). Among these individuals, 33.0% visited the clinics five or more times, and 28.5% filled prescriptions on two or more occasions. Chronic diseases were the second most common reason for onsite clinic visits. The average adherence to prescription as measured by the Proportion of Days Covered (PDC) was 0.61, with 40% of individuals showing a high adherence. Notably, older age and employment at workplaces located outside metropolitan areas were significant factors positively associated with both the utilization of onsite clinic services and adherence to prescribed treatments.

**Conclusion:**

This study found that onsite clinics within a semiconductor company were actively utilized for managing chronic diseases, particularly among older employees and those in workplaces located in areas where medical access is limited compared to metropolitan areas. These findings highlight the potential role of onsite clinics in enhancing chronic disease management. Future research across a broader range of workplaces could further support and expand these insights.

## Introduction

Chronic diseases cause the highest global disease burden, accounting for 41 million deaths annually, which represents 74% of all deaths [1]. Health authorities worldwide are actively working to prevent and control these diseases. In the U.S., the National Center for Chronic Disease Prevention and Health Promotion (NCCDPHP) allocates approximately 1.4 billion dollars annually to prevention programs [2]. Similarly, in South Korea, the National Health Insurance Service (NHIS) offers a general health screening program to prevent and detect chronic diseases, such as hypertension, diabetes, and dyslipidemia, among adult household members, employees, and their dependents [3]. However, the examination rate is only 74.2%, while more than half (58.4%) of the examinees are evaluated to be at risk of diseases [4]. According to World Health Organization, only half of chronic disease patients adhere to their prescriptions [5]. This underscores the need to promote participation in prevention programs and to develop infrastructure at both community and workplace levels for referral and sustainable aftercare [6].

Chronic disease management at community level faces various barriers, including financial challenges, limited access to facilities, and long waiting times for consultations [7]. Furthermore, previous studies have identified additional factors complicating adherence to chronic disease therapy, such as younger age, lower education levels, region of residence, partial payment for treatment, and poor self-perceived health [8,9].

Workplace onsite clinics are emerging as an alternative healthcare system to address these challenges in community healthcare. These clinics have been shown to be cost-effective and offer the potential for more patient-centered care, conveniently located at the worksites, with established communication channels, culture, and support structure [10–12]. Historically, workplace clinics primarily focused on occupational health and minor acute care. However, over the past two decades, they have shifted towards offering preventive services and managing chronic diseases to promote a more productive workforce [11]. A study found that the introduction of onsite clinics reported a significant increase in preventive healthcare utilization, with a 9% increase for female employees and a 14% increase for male employees after implementation [13]. As the National Prevention Strategy has highlighted workplaces as key partners in prevention, worksite health promotion programs (WHPPs) and research on their prevalence and outcomes have expanded recently [14,15]. Nevertheless, the evidence supporting onsite clinics remains less developed compared to the literature on WHPPs [11].

South Korean semiconductor companies present a promising opportunity to study the potential contributions of onsite clinics in managing chronic diseases. Shift work, such as 3-shifts 4-group, is common for non-office workers in the semiconductor industry to maintain continuous operation [16], and has been associated with chronic disease risk [17]. Additionally, most semiconductor employees in Korea over the age of 40 years are male (29.4% versus females 0.7%) [18], a high-risk group for cardiovascular diseases [19]. Given Korea’s significant semiconductor industry, it is feasible to obtain a sufficient number of participants representative of workers at risk of chronic diseases [20]. Moreover, as all employees in South Korean semiconductor companies undergo pre-employment and periodic health examinations, it is relatively easy to secure data for the evaluation of chronic diseases [21].

A leading semiconductor company in South Korea, Samsung Electronics serves as an exemplary model of workplace onsite clinic implementation. Among its facilities, the Kiheng Onsite Clinic operates during regular business hours and is staffed by a team of 22 healthcare professionals including six physicians. All the physicians at the clinic are board-certified specialists in internal medicine, dermatology, psychiatry, and surgery, eliminating the need for referrals from general practitioners and ensuring direct access to expert care for acute, chronic, and mental health conditions, as well as health promotion programs such as smoking cessation and obesity management. To support these programs, the clinic is equipped with a range of medical and diagnostic facilities for such as laboratory tests, X-ray imaging, electrotherapy, and physical therapy [22]. Importantly, this clinic is one of the study sites included in our research.

This study aimed to evaluate the utilization and adherence of onsite clinics and to identify the factors influencing these outcomes, in South Korean semiconductor employees at risk of chronic diseases, including hypertension, diabetes, and dyslipidemia.

## Methods

### Study design

A cross-sectional study was conducted through a retrospective review of electronic medical records (EMR) from worksite onsite clinics. This study was approved by the Institutional Review Board of Seoul National University (IRB No. 1706/002–007, Jun. 9, 2017). Patient consent was waived because all analyses were performed on the company’s secure server using anonymized data for privacy protection.

### Study setting

The study setting was a semiconductor company in South Korea which had three onsite clinics at different workplaces and a total of 39,073 employees as of 2016. Workplace A, located within the metropolitan area, established its onsite clinic in 1989, staffed by five physicians. In contrast, workplaces B and C are located in cities farther from the capital. Their clinics, established in 2005 and 2011, are staffed by three and five physicians, respectively. The onsite clinics provided physician-led primary care services, medications, laboratory tests, and health counseling for internal, surgical, dermatologic, ophthalmic, and psychiatric diseases to employees during normal weekday working hours.

### Study participants

All employees of the semiconductor company were provided with pre-employment and annual periodic health examinations at no cost, in accordance with the Occupational Safety and Health Act in Korea [21]. These examinations were conducted by physicians at hospitals designated as health examination institutions. Based on the results, employees were categorized into health management groups: A, for those in good health; C, for those with suspicious disease; D, for those requiring observation for disease; R, for those needing a follow-up health check-up; and U, for those who did not attend a recommended follow-up check-up [23]. According to the company’s internal standards, employees with a history of chronic disease in their health check-up survey or those classified in the D group were identified as being at risk for chronic diseases, making them eligible for follow-up management [24]. This follow-up management such as health consultation, secondary examination, and treatment, was primarily conducted at the onsite clinics in accordance with the company’s policy.

The participants of this study were the employees at risk of chronic diseases based on health examinations during 2013–2016, who visited the onsite clinics for the management of their chronic conditions. The chronic diseases were defined based on visits with the ICD-10 code of I10* (hypertension), E11* (type 2 diabetes), or E78* (dyslipidemia) in main diagnosis and sub-diagnosis. Employees who joined the company after 2016 or departed before 2016 were excluded from the study due to inadequate data.

### Outcomes and follow-up

We explored the indications for repeated visits to the onsite clinics, the frequency of these visits for chronic disease management, as well as the utilization and adherence to prescription services. Additionally, we investigated the factors influencing the use of prescription services and high adherence to medications.

The indications for repeated visits to the clinics over the study years were defined based on the ICD-10 codes for the main and sub-diagnoses of each visit (S1 Table). The use of prescription services was determined by whether an individual received two or more prescriptions for antihypertensive, antiglycemic, or lipid-lowering agents. Adherence was measured by the proportion of days covered (PDC), calculated as the number of days covered by the prescriptions divided by the total number of days in the period for each patient. A PDC of at least 80% was considered high adherence.

### Statistical analysis

Descriptive statistics, including frequency and percentage, were used to summarize onsite clinic utilization and adherence. To complement this, an analytic study was conducted using multivariable binary logistic regression to estimate adjusted odds ratios (aORs) and 95% confidence intervals (CIs) for identifying factors influencing these outcomes. The confounding variables adjusted for included age group, number of chronic diseases, and disease duration as continuous variables, sex, diagnosis, workplace, onsite clinic, work type, and type of work shift as categorical variables. The shift work system was structured into three shifts—Day (06:00–14:00), Swing (14:00–22:00), and Graveyard (22:00–06:00)—operated under a 3-shifts 4-group schedule [25]. The significance level was set at 0.05. Employees with missing values regarding general and work characteristics were excluded from the analyses. All the analyses were conducted using SAS^®^ 9.4 software (SAS Institute Inc., Cary, NC, USA).

## Results

### Participant characteristics

Among 11,917 visitors to the onsite clinics with abnormal results in health check-ups during 2013–2016, 8,837 employees were evaluated to be at risk of chronic diseases including hypertension (n = 2,239), diabetes (n = 869), or dyslipidemia (n = 7,127). The majority of participants were male (88.2%) and were in their 30s and 40s in terms of age (84.6%). Approximately half of them (56.3%) were non-office workers, and 15.5% were shift workers. Most participants (72.6%) were from Workplace A, a semiconductor facility located within the metropolitan area. The remaining participants were from Workplace B, a semiconductor facility, and Workplace C, a display facility, both located in cities farther from the capital. Detailed characteristics of the participants were presented in Table 1.

**Table 1:** Participant characteristics of the onsite clinic visitors at risk of chronic diseases.

Characteristics		N (%)		
		All visitors (n = 8,837)	Prescription service users^a^ (n = 2,522)	Highly adherent users^b^ (n = 1,016)
**Social demographics**				
Sex	Male	7,791 (88.2)	2,391 (94.8)	981 (96.6)
	Female	1,046 (11.8)	131 (5.2)	35 (59.0)
Age groups	20’s or younger	1,006 (11.4)	73 (2.9)	13 (1.3)
	30’s	4,403 (49.8)	849 (33.7)	272 (26.8)
	40’s	3,071 (34.8)	1,339 (53.1)	594 (58.5)
	50’s or older	357 (4.0)	261 (10.3)	137 (13.5)
**Clinical characteristics**				
Diseases	Hypertension	2,239 (25.3)	1,161 (46.0)	673 (66.2)
	Diabetes	869 (9.8)	435 (17.2)	200 (19.7)
	Dyslipidemia	7,127 (80.6)	1,744 (69.2)	593 (58.4)
Disease duration	1 year	2,682 (30.3)	190 (7.5)	93 (9.2)
	2 years	1,214 (13.7)	277 (11.0)	119 (11.7)
	3 years	2,681 (30.3)	1,072 (42.5)	471 (46.4)
	4 years	2,260 (25.6)	983 (39.0)	333 (32.8)
**Work characteristics**				
Work type	Office work	3,861 (43.7)	1,210 (48.0)	510 (50.2)
	Non-office work	4,976 (56.3)	1,312 (52.0)	506 (49.8)
Type of work shift	Normal working hours	7,454 (84.3)	2,294 (91.0)	943 (92.8)
	Shift working hours	1,366 (15.5)	222 (8.8)	68 (6.7)
	Flexible schedule	17 (0.2)	6 (0.2)	5 (0.5)
Workplace^c^	A (semiconductor industry)	6,416 (72.6)	1,767 (70.1)	645 (63.5)
	B (semiconductor industry)	781 (8.8)	257 (10.2)	152 (15.0)
	C (display industry)	1,640 (18.6)	498 (19.7)	219 (21.6)

^a^Prescription service use determined as receiving two or more prescriptions for antihypertensive, antiglycemic, or lipid-lowering agents.

^b^Highly adherent users defined as 80% or higher proportion of days covered (PDC), calculated as the number of days covered by prescription divided by the number of days in period for each patient.

^c^Workplace A located within the metropolitan area. Workplaces B and C located in cities farther from the capital.

### Indications of the onsite clinic visits

We investigated the indications for 317,430 onsite clinic visits during 2013–2016 by the 8,837 visitors at risk of chronic diseases. The most common indication for onsite clinic visits was rhinitis and acute upper respiratory infection (72,694, 22.9%). Chronic diseases were the second most common indication, with 48,684 cases (15.3%). These included hypertension (20,240, 6.4%), diabetes (5,045, 1.6%), and dyslipidemia (23,399, 7.4%). Other common indications were headache and myalgia (35,452, 11.2%), dyspepsia and gastroenteritis (35,158, 11.1%), and health consultations and vaccinations (20,661, 6.5%) (Table 2).

**Table 2:** Indications of the onsite clinic visits of employees at risk of chronic diseases.

Frequency rank	Number of visits, n (%)	Indication of visits
1	72,694 (22.9)	Rhinitis and acute upper respiratory infection
2	48,684 (15.3)	Chronic diseases (hypertension, diabetes, and dyslipidemia)
3	35,452 (11.2)	Headache and myalgia
4	35,158 (11.1)	Dyspepsia and gastroenteritis
5	20,661 (6.5)	Health consultation and vaccination
6	9,785 (3.1)	Disorders of lacrimal system
7	7,737 (2.4)	Hepatic disease
8	3,177 (1.0)	Tinea pedis
Unranked	84,082 (26.5)	The other Indications
Total	317,430 (100.0)	

### Frequency of the onsite clinic visits for chronic disease management

**Fig 1:**
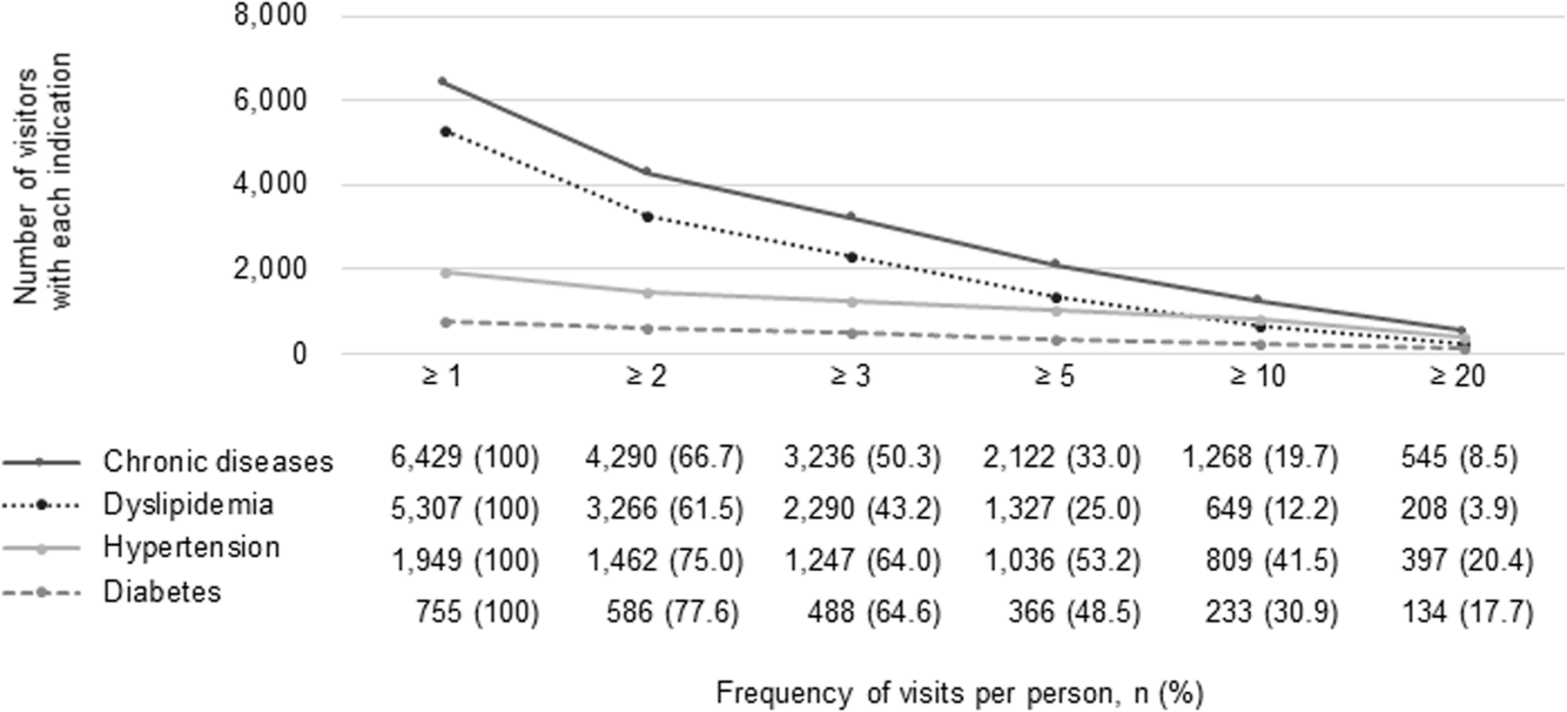
Frequency of the onsite clinic visits for chronic disease management.

We analyzed the frequency of onsite clinic visits for chronic disease management among the 8,837 employees at risk of chronic diseases during 2013–2016.The majority (6,429, 72.8%) utilized the onsite clinics at once for managing their chronic diseases. Within this group, 4,290 employees (66.7%) visited the onsite clinics for chronic disease management twice or more over the study years. Additionally, 2,122 (33.0%) and 1,268 (19.7%) employees had five times or more and 10 times or more visits, respectively, during this period. Onsite clinic visitors with hypertension and diabetes demonstrated higher continuous use compared to those with dyslipidemia (41.5%, 30.9%, and 12.2% visiting ten times or more for hypertension, diabetes, and dyslipidemia, respectively) (Fig 1).

### Prescription service utilization and its influencing factors

Among the 8,837 study participants, 2,522 employees (28.5%) utilized the onsite clinics’ prescription services for their chronic diseases twice or more. The characteristics of prescription service users are detailed in Table 1. In a multivariable binary logistic analysis, several factors were identified as promoting prescription service utilization (Table 3). The presence of hypertension (vs. none) (aOR 4.066, 95% CI 3.530−4.689), diabetes (vs. none) (aOR 2.554, 95% CI 2.165−3.015), increasing disease duration (per year increase) (aOR 1.381, 95% CI 1.308−1.458), older age groups (per decade) (aOR 2.083, 95% CI 1.925−2.255), and being male (vs. female) (aOR 1.578, 95% CI 1.287−1.946) were significant factors. Additionally, working at workplace C, located in a region farther from the capital city, compared to workplace A in the metropolitan area, was also a significant factor (aOR 1.465, 95% CI 1.283−1.672).

**Table 3:** Promoting factors of prescription service utilization and prescription adherence.

Influencing factors	aOR (95% CI)	
	Prescription service utilization^a^	High adherence to prescription^b^
Hypertension (vs. none)	4.066 (3.530–4.689)	4.172 (3.502–4.981)
Diabetes (vs. none)	2.554 (2.165–3.015)	1.743 (1.384–2.196)
Disease duration (per year increase)	1.381 (1.308–1.458)	0.759 (0.693–0.831)
Age group (per decade)	2.083 (1.925–2.255)	1.644 (1.446–1.872)
Male (vs. female)	1.578 (1.287–1.946)	NS
Workplace B (vs. A)^c^	NS	2.255 (1.694–3.013)
Workplace C (vs. A)^c^	1.465 (1.283–1.672)	1.607 (1.289–2.006)

^a^Prescription service use determined as receiving two or more prescriptions for antihypertensive, antiglycemic, or lipid-lowering agents.

^b^High adherence to prescription defined as 80% or higher proportion of days covered (PDC), calculated as the number of days covered by prescription divided by the number of days in period for each patient.

NS, nonsignificant

^c^Workplace A located within the metropolitan area. Workplaces B and C located in cities farther from the capital.

### Prescription adherence and its influencing factors

For the 2,522 users of prescription services, the average prescription adherence was 0.61 ± 0.32. Additionally, 40.0% of these users (1,009 individuals) demonstrated high adherence to their prescriptions, defined by a PDC of at least 80% (Table 4). In a multivariable binary logistic regression, the presence of hypertension (vs. none) (aOR 4.172, 95% CI 3.502–4.981), diabetes (vs. none) (aOR 1.743, 95% CI 1.384–2.196), and increasing age groups (per decade) (aOR 1.644, 95% CI 1.446–1.872) were significant factors associated with a greater proportion of individuals showing high adherence. Furthermore, employees at workplace B and C (vs. workplace A in the metropolitan area) demonstrated higher adherence (aOR 1.607, 95% CI 1.289–2.006 and aOR 2.255, 95% CI 1.694–3.013, respectively). On the other hand, increasing disease duration (per year increase) was associated with lower adherence to prescription (aOR 0.759, 95% CI 0.693–0.831) (Table 3).

**Table 4:** Adherence to prescription in prescription service users.

	Adherence to prescription^a^	Highly adherent users^b^
**Chronic diseases** **(n = 2,522)**	0.61 ± 0.32	1,009 (40.0)
**Hypertension** **(n = 1,161)**	0.74 ± 0.28	673 (58.0)
**Diabetes** **(n = 435)**	0.66 ± 0.31	200 (46.0)
**Dyslipidemia** **(n = 1,744)**	0.55 ± 0.33	593 (34.0)

^a^Adherence to prescription measured in terms of proportion of days covered (PDC), calculated as the number of days covered by prescription divided by the number of days in period for each patient. Data presented as mean ± standard deviation.

^b^High adherence to prescription defined as PDC of at least 80%. Data presented as n (%).

## Discussion

This comprehensive analysis of onsite clinic utilization and adherence highlighted the potential role of workplace clinics, located in areas with limited medical accessibility, for the management of chronic diseases especially among older semiconductor employees. Although most companies provide various health promotion interventions for employees, including drug coverage prescribed by primary clinics, personal training or counseling, few studies have reported on the effectiveness of onsite clinics in delivering comprehensive employees’ healthcare [24,26]. To our knowledge, this research is the first to investigate medication adherence within onsite clinics at a semiconductor company, providing invaluable insights into the integrated role, including drug prescription for chronic disease management within the scholarly research [10].

The analysis of indications for onsite clinic visits demonstrated active utilization of these clinics within the semiconductor company for managing chronic diseases. Chronic diseases accounted for the second most common indication for onsite clinic visits among employees at risk of chronic diseases (15.3% of the total visits, n = 48,684 out of 317,430). This finding is promising, as workplace health promotion programs for workers with chronic diseases have been reported to improve health indicators such as blood pressure, blood glucose and cholesterol levels [24,27]. Considering that these employees use onsite clinics not only for chronic disease management but also for the treatment of various other health conditions, onsite clinics may hold potential as effective primary care centers.

In this study, most employees (99.7%) visited the onsite clinics twice or more for their chronic disease management. Since Korean laws mandate follow-up management for all employees with abnormal health examination results, and the company primarily conducts this management at onsite clinics, most employees at risk for chronic diseases utilize the onsite clinics at a semiconductor company [21,28]. In contrast, reported utilization rates for onsite clinics in other contexts are relatively lower. For instance, a survey by the National Association of Worksite Health Centers and Mercer indicated that only 52% of eligible employees visited onsite clinics in the U.S. in 2020 [29]. Another study reported an average utilization rate of 21.3% for onsite clinics at a beverage company in the U.S. over a four-week period [10]. To enhance onsite clinic utilization rates and prevent the occurrence or worsening of chronic diseases, one potential approach is to encourage companies to establish policies promoting employees access to preventive services at onsite clinics. Establishing such policies may be motivated by the significant association between an increase in the utilization rate and onsite clinic return on investment [10,30].

The study findings indicated that the frequency of onsite clinic utilization differed across disease types. Employees with hypertension and diabetes visited the clinics more continuously than those with dyslipidemia. The onsite medical clinic at 3M implemented a cardiovascular risk reduction program (CVRRP) for employees, significantly lowering lipid levels among participants [27]. Effective management of chronic diseases such as dyslipidemia requires long-term care [31–33]. Given that 80.6% of employees in the semiconductor company were diagnosed with dyslipidemia, targeted interventions are needed to enhance long-term utilization of workplace clinics or community hospitals for this population [33].

In this study, the rate of utilization of prescription services was 28.5%, which was similar to the prescription rate (29%) at the clinics operating CVRRP in 3M [27]. In the long-term management of chronic diseases, adherence to prescribed medications is pivotal to achieve the treatment goal [34]. The average adherence to prescription as measured by PDC was 0.61, which varied by disease, with the highest rates observed in hypertension (0.74), followed by diabetes (0.66) and dyslipidemia (0.55). Adherence for hypertension and diabetes were similar to the PDC (hypertension 0.70–0.80, diabetes and dyslipidemia 0.65–0.75) from an analysis of U.S. insurance data including Medicare and Medicaid [35]. It may be inferred from these results that employees using prescription services for chronic diseases at the onsite clinics consistently utilized the onsite clinics without frequently relying on community healthcare institutions. This suggests that active medication management for chronic diseases is being conducted at the onsite clinics.

In a previous study conducted on employees of the semiconductor company, 74.7% of workers at risk of dyslipidemia had a high 10-year ASCVD risk score [24]. This study showed relatively low medication adherence among visitors diagnosed with dyslipidemia, who accounted for the majority of onsite clinic visitors. Therefore, thorough management, including medication adherence and participation in various other workplace health promotion programs, is necessary for these patients working at the semiconductor company. The high adherence rates ranged from 34–58%, which is lower than the known high adherence rates of 65–75% for chronic diseases [35]. This suggests the potential for a downward adjustment of medication adherence to an average level.

The factors promoting prescription service utilization and high adherence to prescriptions at onsite clinics were older age groups and workplace B or C. The association of better adherence with older age was consistent with a previous study in patients with chronic conditions in primary care [36]. Workplace B and C are located in areas distant from the capital city of Korea, characterized by relatively low population density and limited access to medical facilities. In previous studies, convenience in terms of geographical proximity and time has been reported as the main advantage of onsite clinics [11,12], which can be maximized in these areas with low accessibility to healthcare facilities. Another study reported that treatment adherence positively mediated the relationship between doctor-patient communication and treatment outcomes for rural patients with tuberculosis, compared to urban patients [37]. Therefore, the onsite clinics at workplace B and C could play a role in achieving positive treatment outcomes for workers with chronic diseases through persistent management of medication adherence. A longer disease duration especially in patients with hypertension or diabetes was associated with increased utilization of prescription services at onsite clinics but was also associated with decreased adherence to prescriptions. In general, adherence to long-term medication for chronic diseases remains suboptimal, which may increase the prevalence and complications of these diseases, leading to poor health outcomes and higher healthcare costs [38,39]. Among the chronic diseases analyzed, only dyslipidemia was not associated with higher utilization of onsite clinics, both in terms of prescription service use and adherence. This seems to reflect the relatively lower treatment rate among Korean adults with dyslipidemia compared to those with hypertension and diabetes (hypertension 63.5%, diabetes 59.3%, and dyslipidemia 48.1%) [40]. The determinants associated with non-adherence to the medications for dyslipidemia were reported to include age under 50 years, female gender, smoking habits, being a new user of lipid-lowering medications, and comorbidities such as depression and diabetes [41]. Multifaceted strategies are necessary for workers with dyslipidemia to improve their perception of the importance of adherence, along with the associated potential benefits.

Other occupational variables such as the duration of employment [42,43] and exposures to occupational substances [44] could have been included in the analysis, as they are debated factors in relation to disease occurrence and treatment outcomes in semiconductor employees. Similarly, the number of prescription drugs could also be considered, as it has been reported to lower treatment adherence [45]. However, the duration of employment was highly correlated with younger age, and the number of prescription drugs was strongly associated with the number of diseases, raising concerns about multicollinearity. Regarding occupational exposures, previous studies measuring the biological exposure index (BEI) for substances like ionizing radiation, arsine, benzene, 2-ethoxyethanol, and trichloroethylene in the target workplaces found that most exposure levels were below BEI thresholds, indicating that occupational exposures were unlikely to significantly impact the study results [44]. While these additional variables were excluded for valid reasons, the study results are considered robust. Nevertheless, their potential contributions should not be overlooked in future research.

As in the findings above, this study examined the utilization and adherence to onsite clinic services. Employee satisfaction and perceptions of onsite clinics, which were not reported in this study, have been investigated previously [12]. In a focused-group interview with 72 participants who received chronic disease management at the same onsite clinics, 14% (n=10) believed that onsite clinics could effectively provide chronic disease prevention and management. This perception was attributed to the quality of care and positive support they received from the clinic.

Given the advantages of onsite clinics, the results of this study can serve as a valuable reference for the operation of such clinics, especially in semiconductor companies—the specific setting of this study. Despite the semiconductor industry’s anticipated decade of growth, with an average annual growth rate projected at 6–8% [46], concerns persist regarding the health status of its employees. For instance, shift work, prevalent in the semiconductor sector, is associated with obesity and hypertension, highlighting the urgency of managing chronic health risks for semiconductor employees [16,17]. To enhance the efficacy of onsite clinics in addressing these concerns, previous studies have suggested several improvements, including promoting onsite clinic services and affiliated physicians, providing employee-centered services, and establishing trust in healthcare information privacy [12].

### Strengths and limitations

This study holds significance as it provides evidence on the utilization and adherence of onsite clinics, particularly focusing on promoting factors for chronic disease management. While academic research on this topic is limited, to the best of our knowledge, this is the first investigation specifically addressing medication adherence in onsite clinics within a semiconductor company. Until now, available data has predominantly focused on specific risk reduction initiatives, with most peer-reviewed publications on worksite clinics confined to a single journal [11]. Expanding research on onsite clinics is imperative given the increasing strength of their value proposition. Even amid the Coronavirus Disease 2019 (COVID-19) pandemic, which prompted economic shutdowns and widespread remote work, evidence suggested that workplace health programs, including onsite clinics, were effective in managing employees’ health and yielded a positive financial return on investment [47]. It was reported that during the COVID-19, the onsite clinics offered diagnostic testing for 66% of employees and administered COVID-19 vaccines to 42% of employees. The onsite clinics in each workplace were particularly significant for employees and their dependents who were unable to access in-person healthcare due to infection risks [29]. The advantages of onsite clinics could be maximized in chronic disease management, especially considering that non-occupational medical services provided significantly greater overall savings to employers compared to occupational services (weighted average saving per clinic; occupational $69,758 vs non-occupational $193,857) [10].

This study has several limitations. Since this study did not assess actual treatment outcomes, it cannot evaluate the clinical effectiveness of clinics but focuses instead on their utilization and adherence. The key limitation was the lack of sufficient clinical data for some participants–such as blood pressure, blood glucose, and lipid profiles–which hindered the evaluation of treatment outcomes. Another limitation was the inability to account for essential confounding factors, such as management outside the onsite clinics. This includes treatment in community-based medical institutions and lifestyle modifications through in-house WHPPs, both of which are crucial for assessing overall effectiveness.

The adherence measure utilized in this study has several limitations. The PDC primarily evaluates whether clinic users received their prescriptions over a specified period, reflecting only a limited aspect of adherence from the client’s perspective. It does not account for critical factors influencing adherence, such as service availability or contextual reasons for missed prescriptions, including the resolution of treatment needs or patient oversight. Although more comprehensive tools like the Morisky 8-item scale can assess these aspects [48], the retrospective nature of this study precluded the possibility of conducting new surveys to obtain this data. Despite these limitations, this study made significant efforts to utilize available data to provide valuable insights into the patterns of prescription adherence within onsite clinics. By focusing on a standardized and widely used metric like the PDC, this study offers a consistent and comparable framework for assessing adherence across similar settings. These findings should be interpreted as a reflection of overall adherence behavior within the given context, highlighting potential areas for improvement in service delivery and patient engagement. Furthermore, while the retrospective nature of this study limited the ability to capture behavioral factors, the results still contribute to understanding the utilization and adherence trends of onsite clinic services and their potential role in chronic disease management.

Lastly, it’s important to note that the study focused exclusively on a single semiconductor company in South Korea. In 2018, there were 1,224 semiconductor companies in South Korea employing approximately 165,000 individuals, with 84,751 (50.5%) working in large corporations [49]. This study analyzed data from 39,073 employees within a single semiconductor company, representing 46.1% of the workforce in large semiconductor companies in South Korea. While the findings are limited to one company, they offer valuable insights into the utilization and adherence trends of onsite clinics in a highly structured industrial setting. Given the substantial proportion of the workforce represented in this study, the results may serve as a meaningful reflection of adherence behaviors and service utilization patterns. Furthermore, these findings can provide a basis for exploring similar workplace health programs in other countries with comparable industrial structures and workforce characteristics. Future research involving a broader range of workplaces across various industries and locations will be essential to further validate and generalize these results, enabling a more comprehensive understanding of workplace-based health interventions.

## Conclusion

This study observed that onsite clinics within a semiconductor company were actively utilized for the management of chronic diseases, with chronic conditions being the second most frequent indication and medication adherence levels comparable to those in community healthcare. These findings highlight the potential and direction for the active operation of onsite clinics in chronic disease management, particularly benefiting older employees and those in workplaces with limited access to external medical services demonstrating their potential to address healthcare disparities in the workplace. Future research assessing the effectiveness of onsite clinics through health outcome measurements across various industries and locations could further solidify their role in chronic disease management and their broader impact on workforce health.

## Supporting information

S1 Table List of ICD-10 codes for indications of the onsite clinic visits. (DOCX)
