# Supplementary material for: Onsite clinic utilization and adherence in semiconductor employees at chronic disease risk
Source: PLoS One. 2025 Apr 24;20(4):e0321252. doi: 10.1371/journal.pone.0321252 (PMC12021225; doi:10.1371/journal.pone.0321252)
Supplement: S1 Table — (DOCX) [file pone.0321252.s001.docx]

**S1 Table. List of ICD-10 codes for indications of the onsite clinic visits.**

| **Classification** | **Diagnosis** | **ICD-10 codes** |
| --- | --- | --- |
| Rhinitis and acute upper respiratory infection | Acute nasopharyngitis (common cold) | J00* |
|  | Acute pharyngitis | J02* |
|  | Acute tonsillitis | J03* |
|  | Acute upper respiratory infections of multiple and unspecified sites | J06* |
|  | Acute bronchitis | J20* |
|  | Vasomotor rhinitis | J30* |
| Chronic diseases | Essential (primary) hypertension | I10* |
|  | Type 2 diabetes mellitus | E11* |
|  | Disorders of lipoprotein metabolism and other lipidaemias | E78* |
| Headache and myalgia | Other headache syndromes | G44* |
|  | Dorsalgia | M54* |
|  | Other soft tissue disorders, NEC | M79* |
| Dyspepsia and gastroenteritis | Other gastroenteritis and colitis of infectious and unspecified origin | A09* |
|  | Gastritis and duodenitis | K29* |
|  | Functional dyspepsia | K30* |
| Health consultation and vaccination | Need for other prophylactic measures | Z29* |
|  | Persons encountering health services for other counselling and medical advice, NEC | Z71* |
| Disorders of lacrimal system | Disorders of lacrimal system | H04* |
| Hepatic disease | Other diseases of liver | K76* |
| Tinea pedis | Dermatophytosis | B35* |
| Miscellaneous | The other diagnosis | The other codes |

NEC, not elsewhere classifiable
